# Practical Recommendations in the Treatment of Acute and Chronic Life-Threatening Infectious Diseases in Patients with Acute Hepatic Porphyria

**DOI:** 10.3390/metabo15020099

**Published:** 2025-02-05

**Authors:** Bruno de Mattos Lombardi Badia, Paulo de Lima Serrano, João Paulo Barile, Daniel Delgado Seneor, Patrícia Marques Mendes, Renan Brandão Rambaldi Cavalheiro, Kaliny Oliveira Peixoto, Igor Braga Farias, Roberta Ismael Lacerda Machado, Wladimir Bocca Vieira de Rezende Pinto, Acary Souza Bulle Oliveira, Paulo Sgobbi

**Affiliations:** Division of Neuromuscular Diseases, Porphyria Unit, Federal University of São Paulo (UNIFESP), São Paulo 04039-060, SP, Brazil; bruno.badia@huhsp.org.br (B.d.M.L.B.); barile.joao@unifesp.br (J.P.B.);

**Keywords:** acute hepatic porphyria, infectious disease medicine, anti-infective agents, porphyrins, acute intermittent porphyria

## Abstract

Background: Acute hepatic porphyrias (AHPs) represent inherited metabolic disorders of the heme biosynthesis pathway, leading to neurological and systemic impairment. Despite the presence of well-recognized chronic symptoms and signs, acute neurological, both neuromuscular and central neurological complications pose a significant challenge in clinical practice, with a potential risk of greater severity and mortality during acute decompensation episodes of AHPs. Care related to the prescription of medications, considering the risk of porphyrinogenicity, is a major and recurring concern in the acute and chronic management of AHP patients. Infectious clinical complications are significant issues in both outpatient and hospital settings for patients with AHPs. It is crucial to identify therapeutic regimens with the best safety and efficacy profiles for treating such infectious complications in AHP patients. The scarcity of structured knowledge available in guidelines and recommendations often leads to the use of therapeutic options with higher potential risks in treating patients with AHPs. Objectives: This review article aims to provide practical recommendations for managing the most significant infectious complications in clinical practice, with a focus on their impact on the clinical care of patients with AHPs.

## 1. Introduction

Acute hepatic porphyrias (AHPs) represent a complex group of inherited metabolic diseases secondary to inborn errors of metabolism in the heme biosynthesis pathway [1,2]. Four distinct genetic conditions are included in the AHP group: acute intermittent porphyria (AIP), variegate porphyria (VP), hereditary coproporphyria (HCP), and delta-aminolevulinic acid (ALA) dehydratase (ALAD) deficiency porphyria (or Doss porphyria) [3,4,5]. All forms of AHPs have an autosomal dominant inheritance pattern, except for ALAD deficiency porphyria, with a significant family history for this group of diseases being quite common, despite the incomplete penetrance and variable expressivity observed in the different clinical forms [6,7,8].

Within the typical clinical presentation of AHPs, manifestations in acute neurovisceral crises or acute attacks with intense abdominal pain represent the most feared neurological complications with the greatest potential for lethality, and can often be associated with acute flaccid paralysis due to axonal motor polyneuropathy, acute autonomic dysfunction, intense neuropathic pain in the extremities and, eventually, central nervous system and neuropsychiatric manifestations (including seizures, status epilepticus, acute psychosis, and posterior reversible encephalopathy syndrome) [3,9,10]. In two forms of AHPs, VP and HCP, classic dermatological manifestations associated with photosensitivity can also occur, such as blisters, rash, hypertrichosis, and later scarring and crusting in recurrent lesions. Complications related to impairment by motor axonal polyneuropathy with ventilatory insufficiency and severe dysautonomia, even without the occurrence of central nervous system manifestations, represent the most serious patterns of clinical impairment associated with AHPs [5,11].

The different forms of AHPs can therefore be related to both severe acute complications with a high potential for lethality and chronic complications that are often little known and little valued during clinical follow-up, highlighting systemic arterial hypertension, chronic kidney disease, and the increased risk for hepatocellular carcinoma [12]. There is no mandatory direct correlation between higher rates of acute complications of AHPs or clinical recurrences with the higher rate of chronic complications, although they are more easily identifiable in patients undergoing regular neurological clinical follow-up and who undergo periodic systemic screenings for such chronic contexts. Chronic symptoms and signs related to the disease are also frequent and are commonly attributed to other clinical contexts, such as chronic pain, sleep disturbances, chronic mood disorders, and chronic fatigue, usually leading patients to scenarios of low quality of life and high disease burden [12,13,14]. Therefore, given the entire scenario of chronic manifestations related to the disease, there is no way to limit the view of the context of systemic clinical and neurological manifestations of the different forms of AHPs to isolated acute or merely recurrent presentations.

Both acute and chronic neurological manifestations as well as systemic and dermatological involvement are mostly due to dysfunctions secondary to the toxic effect of intermediate metabolites originating from the biosynthesis pathway of the heme group and porphyrins, with the accumulations of ALA and porphobilinogen (PBG) being those with a greater direct correlation with secondary injuries [15,16,17,18,19]. To a lesser extent, deficits in the formation of the heme group and direct metabolic products of the pathway, such as components of liver P450 cytochromes, myoglobin, catalase, hemoglobin, and peroxidase, may also be involved in the pathophysiology, as well as the greater formation of protein aggregates with potential for proteotoxicity, cellular proteostasis dysfunction, increased occurrence of pro-oxidative mechanisms, and oxidative stress [20,21,22,23].

Different mechanisms have previously been associated with the potential to trigger acute attacks in AHPs, such as alcohol consumption, chronic or acute low carbohydrate intake (e.g., diet, fasting state), toxic triggers (e.g., porphyrinogenic drugs, female hormonal treatments, anesthesia), emotional stress (including lifestyle changes), dehydration, and smoking [10,24,25]. The occurrence of acute infectious diseases represents one of the most common causes of triggering factors for acute neurovisceral crisis and clinical decompensation in patients with AHPs, just as chronic infectious contexts may represent an additional mechanism for difficulty in symptomatic control of patients with AHPs, especially in scenarios that require chronic or prolonged use of antimicrobial agents or that may be related to secondary clinical complications [19]. Likewise, both acute systemic infections and chronic infectious contexts may require prolonged use of potentially porphyrinogenic drugs [26].

A few articles, guidelines, and recommendations have been developed and proposed to aid clinicians in their decision making in real-world setting. The objective of this article is to present the current knowledge base related to care, practices and recommendations for patients with AHPs who present some type of acute or chronic systemic infectious complication that requires therapeutic drug interventions.

## 2. Drug Prescription in the Context of AHPs

### 2.1. Mechanisms Associated with the Porphyrinogenic Potential of Drugs

The restrictions related to drug prescription in several acquired and hereditary neuromuscular diseases and inherited metabolic disorders represent a significant challenge in clinical practice. They may result from various mechanisms where different dysfunctions, either directly or indirectly, lead to clinical or laboratory worsening or progression. The most well-known example of disease exacerbation or clinical worsening through drug exposure is represented by autoimmune acquired myasthenia gravis, a condition involving primary immune-mediated dysfunction with impairment of the neuromuscular junction. In the context of this autoimmune neuromuscular disease, there is both the possibility of severe myasthenic exacerbation or myasthenic crisis following drug exposure in patients already diagnosed with the disease, as well as rare clinical contexts of drug-induced forms (e.g., immune checkpoint inhibitors, D-penicillamine) [27]. Given the large number of drugs with potential harm to the postsynaptic membrane of the neuromuscular junction, there is a recommended list of contraindicated drugs that is regularly reviewed and updated. This list is now more widely recognized by clinicians across different settings (emergency, intensive care unit, outpatient clinics) [27]. For the described context of autoimmune myasthenia gravis, no exacerbations directly related to known pharmacogenetic factors have been described. However, direct toxic effects on peripheral nerves also represent a potentially harmful mechanism in patients with the most common hereditary neuropathy worldwide, known as Charcot–Marie–Tooth disease. In these cases, both axonal and demyelinating forms are associated with risks following exposure to various drugs, especially antineoplastic chemotherapeutic agents (e.g., vincristine, paclitaxel) [28].

In the case of inherited metabolic disorders, such as several primary mitochondrial diseases, there are also formal recommendations for avoiding certain drugs. For example, medications with toxic potential for different mitochondrial functions should be avoided in cases of primary mitochondrial myopathies [29,30]. In some well-known scenarios, such as in *POLG*-related disorders, for example, one of the most commonly prescribed antiepileptic drugs, valproate, is absolutely contraindicated, as well as in several different mitochondrial DNA-related disorders and nuclear DNA-associated mitochondrial diseases, several drugs have been demonstrated to be harmful due to different mechanisms, including inhibition of mitochondrial ribosomal protein synthesis and disruption of mitochondrial oxidative phosphorylated system [29]. Several complications have been observed after drug exposure in these scenarios, such as acute liver failure, rhabdomyolysis, toxic myopathy, and propofol infusion syndrome [29,30]. Currently, there are consensus guidelines that assist clinicians and researchers in understanding the best safety profiles for treating patients with mitochondrial diseases [31,32]. A similar need for restrictive care in certain aspects of medication use and dietary management can also be observed in metabolic myopathies that lead to potentially severe acute complications, such as rhabdomyolysis with myoglobinuria [33,34].

The decision regarding different therapeutic schemes to be employed in the treatment of acute and chronic conditions represents one of the most critical steps in the clinical management of patients with AHPs [35,36]. One of the most important mechanisms correlated with the potential porphyrinogenic deleterious effects of certain drugs is related to their capacity to induce a higher activity of cytochrome P450 enzymes, which are fundamental elements in hepatic drug metabolism and in the binding of proteins to the heme group, a product of the biosynthesis pathway of protoporphyrin and heme group [37,38,39,40,41]. Thus, contact with drugs that have the potential to induce a higher heme biosynthesis for cytochrome P450 and, consequently, an increased demand on the metabolic pathway involving defective enzymes in the various forms of AHPs, results in greater stimulation of a pathway with enzymatic deficiencies. This can lead to the accumulation of toxic intermediates, such as ALA and PBG, with the potential to trigger acute attacks accompanied by acute neurovisceral crisis. It is important to highlight that some *CYP2D6* polymorphisms may also play a role in the penetrance of AIP, and then, an individual genetic basis factor can also represent a key mechanism in porphyrinogenicity [42]. There are, however, additional modulatory factors influencing this pathway, such as those associated with increased induction of ALA synthase-1, secondary effects of drugs with the potential to inactivate or inhibit cytochromes, and the induction of heme oxygenase-1 expression, the primary enzyme involved in heme catabolism [37,38,39].

The different mechanisms which are involved with neurotoxic mechanisms in AHPs may also be involved with other pathways seen in drug porphyrinogenicity and in an AHP trigger: (i) ALA-related neurotoxicity leading to direct neurotoxicity, gamma-aminobutyric agonism, peptide transporter 2-mediated neurotoxicity, autooxidation, and dysfunction of voltage-gated sodium channels; and (ii) heme deficiency may lead to mitochondrial dysfunction and oxidative stress, nitric oxide/cyclic guanosine monophosphate pathway failure, increased serotonin, and gamma-aminobutyric acid deficiency [23]. The porphyrinogenic potential may also be related secondarily to the impact of mechanisms that increase the demand on the heme biosynthesis pathway (e.g., in the context of recurrent or chronic hypoglycemia), to endocrine porphyrinogenic effects (e.g., androgenic or progestagenic effects), or to precursors or intermediate metabolites resulting from secondary metabolic pathway deviations induced by various drugs [37,43,44].

**Figure 1:**
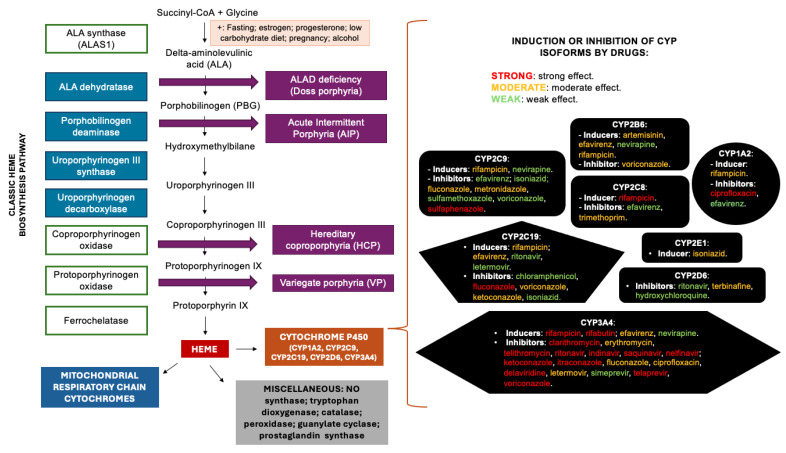
Summary of the main steps involved in heme biosynthesis, the main products (hemeproteins) which result from heme use, and the porphyrinogenic risks involved with the use of antimicrobial agents in patients with AHPs and their association with the potential induction or inhibition of cytochrome P450 isoforms. On the left side of the figure, in the heme biosynthesis pathway, intramitochondrial enzymes and steps are represented inside light blue squares. Each of the AHP subtypes are highlighted in purple squares related to the corresponding defective metabolic step [5,20]. On the right side of the figure, each black diagram represents one subtype of cytochrome P450 isoform and its related inductors and inhibitors. The increased need of each isoenzyme after drug induction leads to a higher need of heme content and increased biosynthesis pathway activation, resulting in a higher porphyrinogenic risk from the defective pathway in different AHP types due to raised levels of neurotoxic ALA and PBG production. Drugs with strong effects on inhibition or induction for each isoform are represented with red letters. Drugs with moderate effects are represented with yellow letters. Drugs with weak effects are represented with green letters. Data related to cytochrome P450 isoenzymes induction or inhibition have been checked in an updated reference: Cytochrome P450 Drug Interactions Flockhart Table^TM^, Clinical Pharmacology Research Institute, Department of Medicine, Indiana University. Available online: URL: https://drug-interactions.medicine.iu.edu/MainTable.aspx (last accessed on 7 January 2025) [38,39,47]. Legends: AIP: acute intermittent porphyria; ALA: delta-aminolevulinic acid; ALAD: ALA dehydratase; ALAS1: ALA synthase 1; HCP: hereditary coproporphyria; NO: nitric oxide; PBG: porphobilinogen; VP: variegate porphyria; +: symbol for factors which stimulate ALAS1 activity.

Several methods, markers, guidelines, and techniques have been developed to improve the potential for prevention and identification of porphyrinogenic risks associated with certain drugs. These models and methods represent some of the most commonly used approaches in predictive algorithms for the development of drug safety profile lists [40,41,43,45], which are discussed in greater detail in Section 2.3 of this article. However, there is still a limited and often imprecise correlation between data obtained in isolation from animal models or cell and tissue cultures and observations derived from small clinical case reports or case series in the medical literature [37]. Similarly, the role of individual pharmacogenetic markers related to cytochrome P450 components (e.g., *CYP2D6* gene polymorphisms) remains poorly understood and established, particularly regarding their significance and clinical impact. This is especially relevant when considering the measures required for treating AHPs and making decisions about the continuation or discontinuation of drugs with varying degrees of porphyrinogenic risk [44,46]. Figure 1 summarizes the heme biosynthesis pathway and the porphyrinogenic potential and risks related to antimicrobial agents used in the treatment of infectious diseases in patients with AHPs and their association with the induction or inhibition of different cytochrome P450 isoforms by these drugs.

### 2.2. Differentiation Between Adverse Drug Event and Symptomatic Exacerbation of AHPs

An adverse event represents any type of medical occurrence (e.g., an unintended clinical sign or symptom) involving a patient or a clinical trial subject using an investigational product or drug, regardless of the severity, outcomes, or potential causal relationship with the used or studied drug. Not all adverse events within the context of clinical care or clinical trials result in greater clinical severity nor are they directly related to the exposed drug [48]. Nonetheless, serious adverse events (SAEs) represent those adverse events which occur after any drug dose exposure resulting in death, in life-threatening complications leading to hospitalization (for at least 24 h in duration or in a context with an evident need for emergency care) or prolongation of previous hospitalizations, in persistent significant disability, or in congenital anomalies or birth defects in pregnancy-related contexts. All SAEs need to be immediately reported at the latest within 24 h after the clinician awareness of the events to the Sponsor and Institutional Review Board during clinical studies and to the Biovigilance systems from the regulatory health authorities, national pharmacovigilance centers, or to the drug manufacturer during routine clinical setting [49]. Moreover, unlike adverse events, side effects represent outcomes distinct from the primary effect or therapeutic mechanism related to a drug’s action. In some clinical contexts, they may even represent a desired or beneficial effect within the proposed treatment [50].

Adverse drug reaction (ADR) represents undesirable and unintended effects observed after an individual is exposed to a drug (either from a single dose or prolonged exposure). These reactions can result from a direct toxic mechanism, na adverse response through a pharmacological mechanism, an immune-mediated reaction, or an idiosyncratic response of the individual [51,52]. Serious adverse drug reactions (SADRs) represent those SAEs which are considered to be directly related to the drug treatment and are more commonly unexpected. Even with the severity and great complexity involved in different types of SAEs, most of the new knowledge in this context results from pharmacovigilance studies, systematic reviews/meta-analyses and, more rarely, reports to local or national public databases [52]. Pharmacogenetic markers are increasingly being recognized as potential biomarkers associated with a higher susceptibility to drug-specific adverse drug reactions [52]. Different specific criteria and algorithms for assessing the potential causality in the occurrence of adverse drug reactions have been developed over the last few decades. Notably, the Naranjo algorithm and the World Health Organization-Uppsala Monitoring Center (WHO-UMC) criteria have contributed over time to a greater understanding of the causality of adverse drug reactions [53].

In outpatient clinical practice or emergency service settings for patients with AHPs, it is often challenging to differentiate whether a symptom presented as a possible adverse event is solely related to the medication used or to a potential worsening or exacerbation of AHPs. Some common adverse event scenarios, such as abdominal pain and gastrointestinal symptoms, like diarrhea and constipation, can be especially complex in this type of differential diagnosis assessment. It is essential for clinicians to identify other clinical signs and symptoms (e.g., limb muscle weakness, signs of dysautonomia, suggestive photosensitive skin lesions, acute neuropsychiatric disturbances, and sleep–wake disorders) or laboratory findings (e.g., elevation of delta-aminolevulinic acid and porphobilinogen in urine samples) that could support the identification of causes related to acute metabolic decompensation [5,9].

Establishing an early differentiation between these scenarios or their potential overlap is a difficult task, but one that leads to different clinical management strategies. These may include the potential suspension or discontinuation of drugs that are potentially harmful or pose a higher porphyrinogenic risk, replacement with active ingredients that have a better safety profile for patients with AHPs, closer clinical and laboratory monitoring for patients experiencing recent acute exacerbation, and the implementation of preventive clinical measures (e.g., reserving hospital admission slots or monitoring in an intensive care unit or under hemin therapy in cases of clinical progression).

### 2.3. Safe and Unsafe Drug Lists

Updated and revised lists of medications aim to promote safer, individualized treatments, avoiding unnecessary therapeutic approaches or those with potential for acute exacerbations or inadvertent clinical worsening with higher risks of complications, hospitalization, or even death. These lists aim to provide guidance on the current state of the art regarding the drug-induced risk of triggering acute neurovisceral crisis or contributing to clinical or laboratory deterioration. There are numerous clinical contexts in which the safety profile of certain medications remains undefined or uncertain, especially when there is a lack of information from medical reports in scientific articles or from reports during pharmacovigilance activities or to national and international regulatory agencies. It should be emphasized that, currently, there is no scientific evidence demonstrating a poor safety profile for the use of any type of approved human immunization in patients diagnosed with AHPs.

In this article, healthcare-related infectious disease or nosocomial acquired infectious conditions, such as ventilator-associated pneumonia and surgical wound infections, will not be discussed in detail due to the higher specificity related to different institutional protocols, the greater need for individualized treatments (e.g., considering local antimicrobial resistance profiles), and the lower demand compared to outpatient or urgent/emergency settings.

It is important to emphasize that the content expressed in this article does not substitute the need for individualized medical evaluation for each clinical context presented in clinical practice, nor does it constitute an isolated element of professional advice or represent a restriction of limitation on the prescribing physician. The authors also highlight that treatment decisions should take into account local and national clinical guidelines, as well as aspects related to good prescribing clinical practices and respecting the autonomy of the attending physician. Any pharmacological treatment option adopted for patients with AHPs is the sole responsibility of the attending physician. Furthermore, it is not recommended for patients diagnosed with AHPs to make any type of treatment decision based on self-medication practices, and it is strictly necessary to carry out a formal medical evaluation followed by the corresponding therapeutic purposes from the attending physician.

Given the significant expansion and frequent updates of new knowledge related to pharmacology in the treatment of rare diseases and pharmacovigilance aspects, it is highly recommended that information databases and data sources with periodic and continuous medical updates be consulted in addition to the content presented in this review, with important references highlighted in the topic of AHPs: (i) Drug Database from the American Porphyria Foundation (APF) (free access available at URL (accessed on 10 December 2024): https://porphyriafoundation.org/drugdatabase/) [54]; (ii) Acute Porphyria Drug Database, established and maintained by The Norwegian Porphyria Centre (NAPOS), from the Haukeland University Hospital, in Norway (free access available at URL (accessed on 10 December 2024): https://drugsporphyria.net/clear/1) [55]; and (iii) Safe List from the United Kingdom Porphyria Medicines Information Service (UKPMIS) and Cardiff Porphyria Service (at the University Hospital of Wales), supported by the National Acute Porphyria Service (NAPS) (free access available at URL (accessed on 10 December 2024): https://www.wmic.wales.nhs.uk/specialist-services/drugs-in-porphyria/) [56].

Based on the classification of the safety profile of different active principles in patients with AHPs, according to the Acute Porphyria Drug Database (The Norwegian Porphyria Centre/NAPOS; available at URL (accessed on 10 December 2024): https://drugsporphyria.net/riskclasses) [55], six distinct classes can be distinguished regarding porphyrinogenicity: (i) “Unknown/not yet classified”: drugs that should be avoided due to few safety conclusion—used only if no additional alternatives are available; (ii) “Porphyrinogenic”: drugs with well-proven correlation with porphyrinogenic potential, which should only be used on emergency/urgency settings and with specific and general precautions for AHPs; (iii) “Probably porphyrinogenic”: drugs with high risk of porphyrinogenicity and used only on urgency/emergency settings and with special care and attention; (iv) “Possibly porphyrinogenic”: drugs with associated risk which should only be used in case of no additional safe second-line choice of treatment; (v) “Probably not porphyrinogenic”: drugs with good safety profile and probably low risk with no need for specific care for AHPs; and (vi) “Not porphyrinogenic”: drugs with well-established good safety profile and with no need for specific precautions for AHPs [55].

## 3. Treatment of Acute Bacterial Infectious Diseases

### 3.1. Community-Acquired Pneumonia (CAP)

Community-acquired pneumonia (CAP) represents one of the infectious diseases with the highest potential severity in cases of delayed diagnosis and inappropriate treatment. In the clinical context of outpatient treatment for patients without a previous diagnosis of AHPs, the use of empirical treatment with amoxicillin, doxycycline, or macrolides, such as clarithromycin and azithromycin, is recommended. For outpatients with clinical comorbidities other than AHPs, amoxicillin/clavulanic acid or a cephalosporin (cefuroxime) combined with a macrolide or doxycycline, or a respiratory fluoroquinolone (levofloxacin, moxifloxacin) may be used. It is essential to carefully assess the presence of risk factors for antimicrobial resistance, as well as to stratify the risk for cases requiring hospitalization. In hospitalized cases outside the Intensive Care Unit (ICU) context, empirical treatment may include a respiratory fluoroquinolone (levofloxacin, moxifloxacin) or a combination of ampicillin/sulbactam or cephalosporin (ceftriaxone, cefotaxime) with doxycycline or a beta-lactam combined with macrolides (azithromycin, clarithromycin). If there is a risk factor for methicillin-resistant *Staphylococcus aureus* (MRSA) infection, the addition of vancomycin or linezolid is necessary. In cases of risk for *Pseudomonas aeruginosa*, the addition of piperacillin/tazobactam, cefepime, ceftazidime, meropenem, or imipenem/cilastatin is required. For ICU-admitted cases with severe respiratory infections, intravenous empirical treatment is indicated, potentially including the combination of beta-lactams (ampicillin/sulbactam) or cephalosporins (cefotaxime, ceftriaxone) with macrolides (clarithromycin, azithromycin) or beta-lactams with respiratory fluoroquinolones (levofloxacin, moxifloxacin) [57,58]. In cases with positive concurrent testing for influenza, antiviral treatment (oseltamivir) is recommended, as discussed in Section 9 of this article.

In the treatment of outpatient cases in patients with AHPs, the use of amoxicillin and amoxicillin/clavulanic acid is considered non-porphyrinogenic and safe, while azithromycin is probably non-porphyrinogenic, and clarithromycin is possibly porphyrinogenic. In such cases, it is important to establish the severity of the clinical presentation, the presence of clinical comorbidities, and the stability of AHPs. Both azithromycin and amoxicillin are suitable options, presenting a good safety profile without significant risks of complications. For cases with additional risk factors in patients with AHPs, the combination of amoxicillin/clavulanic acid or cefuroxime with azithromycin or a respiratory fluoroquinolone (such as levofloxacin or moxifloxacin), which are considered probably non-porphyrinogenic, may be proposed. Further details on the porphyrinogenic profile of commonly used antimicrobial agents can be found in Table 1.

### 3.2. Acute Otitis Media and Bacterial Sinusitis

Most clinical contexts of acute otitis media are managed on an outpatient basis, with intravenous in-hospital treatment recommended for cases associated with severe clinical complications (e.g., mastoiditis, septic thrombophlebitis of dural sinuses) or requiring surgical intervention. For cases where outpatient antibiotic therapy is indicated in patients without AHPs, the drug of choice is amoxicillin. Alternatives include amoxicillin/clavulanic acid, cephalosporins (cefuroxime), or macrolides (clarithromycin, azithromycin) in cases of amoxicillin resistance [59,60]. Considering the safety profile in patients previously diagnosed with AHPs, amoxicillin or amoxicillin/clavulanic acid, which are considered not porphyrinogenic, can be used for outpatient treatment of acute otitis media. In cases with potential resistance to amoxicillin, cefuroxime or azithromycin, which are classified as probably not porphyrinogenic, may also be considered.

In cases of suspected uncomplicated acute bacterial rhinosinusitis in patients without AHPs, amoxicillin or amoxicillin/clavulanic acid can be used. In cases of penicillin allergy, cefuroxime is an option, while for beta-lactam allergies, macrolides (clarithromycin), doxycycline, or respiratory fluoroquinolones (levofloxacin) may be considered. General measures should also be implemented, such as nasal irrigation with saline solution, the use of topical nasal corticosteroids (e.g., budesonide, fluticasone), and decongestants (e.g., pseudoephedrine, cromoglicic acid, chlorpheniramine) [61,62,63]. For cases of acute bacterial sinusitis in patients previously diagnosed with AHPs, amoxicillin or amoxicillin/clavulanic acid can be used and represent the first choice of therapy, while, in cases associated with allergies or additional mechanisms of resistance, cefuroxime and respiratory fluoroquinolones, such as levofloxacin, may be used.

### 3.3. Cellulitis and Erysipelas

Among the main skin and soft tissue infections, erysipelas is the most commonly associated with *Streptococcus pyogenes*, while cellulitis is often linked to *Staphylococcus aureus* infection. Erysipelas involved infectious compromise of the superficial layers of the epidermis, whereas cellulitis affects deeper tissue layers. Uncomplicated erysipelas can be treated with empirical oral antibiotic therapy, including amoxicillin/clavulanic acid, cefadroxil, or clindamycin. More severe cases of cellulitis and erysipelas may require intravenous treatment with oxacillin, clindamycin, or cefazolin. In diabetic patients, outpatient cases are recommended to be treated with a combination of ciprofloxacin and clindamycin, while hospitalized cases are managed with an intravenous combination of ceftriaxone and clindamycin [64]. For patients with a history of intravenous drug use, in-hospital intravenous treatment is recommended, including the combination of piperacillin and vancomycin. In severe cases, such as necrotizing fasciitis, intravenous ceftriaxone and clindamycin are used, along with concurrent surgical evaluation. For tropical pyomyositis, surgical drainage and intravenous oxacillin are the treatments of choice. Purulent infections with abscesses require surgical drainage and systemic antibiotic therapy, which may include intravenous oxacillin, cefazolin, or clindamycin [64].

In patients with a prior diagnosis of AHPs, uncomplicated erysipelas can be treated with amoxicillin/clavulanic acid and cefadroxil. Clindamycin, due to its probable porphyrinogenic profile, should not be routinely used in outpatient cases without additional risk factors. For more severe cases of cellulitis and erysipelas in AHP patients, the use of oxacillin and clindamycin should be cautious and considered for patients requiring hospitalization, receiving special care and attention due to the potential porphyrinogenic profile. In other contexts, the use of ciprofloxacin and ceftriaxone, considered probably not porphyrinogenic, and piperacillin and vancomycin, with a not porphyrinogenic profile, may be indicated depending on the need of in-hospital settings for patients with a history of intravenous drug use or in scenarios involving polymicrobial flora. Further details on the porphyrinogenic profile of commonly used antimicrobial agents can be found in Table 1.

### 3.4. Urinary Tract Infections (UTIs)

In patients without a diagnosis of AHPs, uncomplicated UTIs may be treated with amoxicillin/clavulanic acid or cephalosporins (cefuroxime, cefadroxil), with ciprofloxacin as an alternative in cases of allergies. For cases requiring in-hospital intravenous treatment, ceftriaxone or cefotaxime is indicated, with amikacin or piperacillin-tazobactam as alternatives for allergic patients [65]. In patients with a prior diagnosis of AHPs presenting with uncomplicated UTIs, amoxicillin/clavulanic acid, cefuroxime, and cefadroxil are suitable options due to their relatively safe profiles. Ciprofloxacin is also considered probably not porphyrinogenic. For in-hospital treatments of UTI in AHP patients, ceftriaxone or cefotaxime, which are probably not porphyrinogenic, may be used. Additionally, amikacin (not porphyrinogenic) and piperacillin-tazobactam (probably not porphyrinogenic) can be considered in appropriate cases.

### 3.5. Bacterial Infections of the Gastrointestinal Tract

In the context of bacterial infections of the gastrointestinal tract, the key aspects to consider in therapeutic decision-making include prior identification of the associated infectious agent, the site of gastrointestinal involvement, and the clinical severity. For the treatment of *Helicobacter pylori* infections (most commonly associated with peptic ulcer disease), in patients without AHPs, a combination of clarithromycin and amoxicillin or metronidazole is recommended, along with the concomitant use of proton pump inhibitors (e.g., omeprazole, lansoprazole). In cases of bacterial diarrhea caused by *Campylobacter* or *Shigella*, in patients without AHPs, the use of quinolones (ciprofloxacin) or azithromycin may be employed, as well as ceftriaxone or cefotaxime in more severe infections. For infections caused by *Escherichia coli*, both ciprofloxacin and cotrimoxazole (sulfamethoxazole-trimethoprim) can be used [66]. In patients without AHPs, infections caused by *Clostridium difficile*, such as pseudomembranous colitis, can be treated with oral metronidazole or vancomycin [67]. In cases of acute cholangitis and cholecystitis (including common pathogens, such as *E. coli*, *Klebsiella* spp., and *Enterococcus* spp.), in patients without AHPs, a combination of ceftriaxone or cefotaxime with metronidazole may be used. This regimen can also be applied in uncomplicated appendicitis. For gastrointestinal infections caused by anaerobic bacteria (e.g., *Bacteroides fragilis*), in patients without AHPs, metronidazole or clindamycin may be used in mild to moderate cases, while severe forms may require piperacillin-tazobactam or carbapenems (meropenem, imipenem) [68].

Considering the context of patients with AHPs, various therapeutic considerations can be proposed. In *Helicobacter pylori* infections in patients with AHPs, amoxicillin can be used in combination with clarithromycin, provided there is good prior control of AHPs and close clinical monitoring for signs of acute exacerbation. Metronidazole can be used under strict clinical monitoring in cases requiring therapeutic combination. Among the associated proton pump inhibitors, pantoprazole is preferred due to its better safety profile. In cases of acute bacterial diarrhea caused by *Campylobacter* or *Shigella* in patients with AHPs, ciprofloxacin, azithromycin, ceftriaxone, or cefotaxime can be used due to their probably not porphyrinogenic profiles. For gastrointestinal infections caused by *E. coli* in patients with AHPs, ciprofloxacin can be used, while sulfamethoxazole-trimethoprim should be avoided due to its porphyrinogenic profile. For pseudomembranous colitis in patients with AHPs, oral vancomycin or oral fidaxomicin, both with not porphyrinogenic profiles, can be used. In severe or refractory cases, where absolutely necessary, metronidazole, which has a possibly porphyrinogenic profile, may be used as an adjunct under careful clinical and laboratory monitoring. In mild to moderate cases of acute cholecystitis or cholangitis in patients with AHPs, amoxicillin/clavulanate, ceftriaxone, or cefuroxime can be used. In severe contexts or cases with a risk of multidrug resistance, piperacillin/tazobactam, amikacin, or meropenem can be used. For infections caused by anaerobic bacteria in patients with AHPs, amoxicillin/clavulanate can be used for mild cases, while piperacillin/tazobactam and meropenem are options for severe forms due to their good safety profiles. On the other hand, the use of clindamycin, possibly porphyrinogenic, should be reserved for severe cases and performed under clinical and laboratory monitoring due to the higher associated risk of porphyrinogenicity. Further details on the porphyrinogenic profile of commonly used antimicrobial agents can be found in Table 1.

### 3.6. Bacterial Gynecological Infections

The most clinically significant gynecological infectious context is represented by acute pelvic inflammatory disease (PID). The most common infectious agents in this polymicrobial condition include *Neisseria gonorrhoeae*, *Mycoplasma hominis*, and *Chlamydia trachomatis*, although various other agents may also be involved. This scenario includes presentations, such as endometritis, salpingitis, and tubo-ovarian abscesses, with or without associated peritonitis. Upon clinical suspicion, immediate early empirical treatment is recommended. In forms without peritonitis, a combination of ceftriaxone or cefotaxime, doxycycline, and metronidazole can be used. In forms with peritonitis, a combination of ceftriaxone, metronidazole, and doxycycline; clindamycin with gentamicin; or ampicillin/sulbactam with doxycycline can be administered, with doxycycline or clindamycin maintained after hospital discharge. In cases of intact tubo-ovarian abscesses, the antimicrobial regimen is similar to that used in forms with peritonitis. For ruptured abscesses, surgical drainage is required, and the antimicrobial regimen is similar to that used for intact abscesses [69,70,71].

In patients with AHPs, ceftriaxone, cefotaxime, doxycycline, and metronidazole can be used for forms without associated peritonitis, with preference for the first three agents. For forms with associated peritonitis, patients with AHPs may use ceftriaxone, doxycycline, and metronidazole, or in more severe cases, ampicillin/sulbactam with doxycycline or gentamicin with clindamycin, provided that careful clinical and laboratory monitoring is conducted. Further details on the porphyrinogenic profile of commonly used antimicrobial agents can be found in Table 1.

### 3.7. Acute Bacterial Meningitis

The empirical treatment regimen with antimicrobials is recommended until a specific etiological agent has been identified, with the intravenous use of ceftriaxone and vancomycin being indicated. The initiation of dexamethasone use in all patients before the first dose of antibiotic therapy is recommended. In individuals with beta-lactam allergies, meropenem can be used. In cases where agents such as *Haemophilus influenzae* and *Neisseria meningitidis* have been identified, ceftriaxone is indicated, with cefepime as an alternative for *Haemophilus influenzae* and meropenem as an alternative for *Neisseria meningitidis*. In cases of *Streptococcus pneumoniae* isolation, ceftriaxone and vancomycin are indicated, with meropenem as an alternative combination. In cases of listeriosis, intravenous ampicillin is indicated, with alternatives being sulfamethoxazole/trimethoprim or meropenem. In cases of oxacillin-sensitive *Staphylococcus aureus*, intravenous oxacillin can be used, with linezolid or daptomycin as alternatives, while for oxacillin-resistant strains, vancomycin is the drug of choice [72].

In patients with AHPs, within the initial therapeutic regimen, ceftriaxone or cefotaxime can be used, as well as vancomycin for cases at risk of *Streptococcus pneumoniae* resistance, or ampicillin for cases at increased risk of *Listeria monocytogenes*. The use of alternatives in listeriosis cases, such as meropenem, may be considered, while the use of sulfamethoxazole/trimethoprim should be avoided. Table 1 summarizes the main data related to the safety profile of different drugs used in the treatment of acute bacterial infections in patients with AHPs.

## 4. Treatment of Mycobacterial Infections: Tuberculosis and Leprosy

The treatment of leprosy in chronic infections by *Mycobacterium leprae* depends on the multibacillary or paucibacillary profile of the disease. For paucibacillary forms, the use of rifampicin and depose for 6 months is recommended, while for multibacillary forms, the treatment involves a single multidrug therapy, including rifampicin, dapsone, and clofazimine, for 12 months. In cases of rifampicin resistance, it may be replaced by minocycline, ofloxacin, or clarithromycin, depending on whether there is resistance to ofloxacin [73]. The treatment of pulmonary tuberculosis in non-resistant *Mycobacterium tuberculosis* infection should include the use of rifampicin, isoniazid, pyrazinamide, and ethambutol for 2 months, followed by rifampicin and isoniazid for an additional 4 months. In cases of multidrug-resistant tuberculosis (MDR-TB), prolonged therapy for 18 to 24 months with an alternative therapeutic regimen may be necessary, including drugs such as levofloxacin, amikacin, clofazimine, and linezolid. Cases associated with extensively drug-resistant tuberculosis (XDR-TB) should preferably be monitored and managed by specialized tuberculosis treatment teams [74]. Other specifics related to the treatment of tuberculosis in patients with chronic HIV/AIDS infections are discussed in Section 7 of this article. The occurrence of adverse events commonly associated with the different components of antimicrobials in the treatments of tuberculosis and leprosy always represents a major challenge in clinical practice.

In the context of tuberculosis treatment in patients with AHPs, it must be well recognized that most drugs with the best clinical response profile for tuberculosis also have a higher potential to be porphyrinogenic inducers. In various instances in the medical literature, patients have been reported with either the first clinical decompensation of AHP after starting treatment with anti-tuberculosis drugs or in patients previously diagnosed with AHP [75,76,77]. According to The Porphyria Information Centre at the University of Cape Town [78] (available at: URL (accessed on 10 December 2024): https://porphyria.uct.ac.za/porphyria-professionals/porphyria-professionals/prescribing-porphyria-treatment-specific-disorders-poprhyria/treatment-tuberculosis-patients-porphyria), the increased risk ratios are associated with drugs such as rifampicin, rifabutin, ethionamide, and pyrazinamide, as well as the monitored and careful use recommended for ofloxacin and isoniazid. The risks are significantly lower with the use of drugs such as ethambutol, streptomycin, kanamycin, and amikacin. As discussed earlier, additional care is always recommended to provide hemin therapy as a rescue treatment for patients who require anti-tuberculosis treatment with porphyrinogenic drugs. In the case of first-line anti-tuberculosis drugs, it is recommended to start with isoniazid, followed by rifampicin or rifabutin, and then ethambutol, and, more rarely, if the patient adapts well to all drugs, pyrazinamide. In the cases of second-line alternative agents, outpatient streptomycin or amikacin in hospitalized cases is recommended, combined with ethambutol and ofloxacin [78]. If clinical decompensation with acute neurovisceral crisis occurs in a patient with AHP treated for tuberculosis, rescue therapy with hemin is recommended, along with the suspension of therapy during the acute phase. Only ethambutol and streptomycin or amikacin may be used, and ofloxacin should be introduced later, once neurological stability is achieved, with isoniazid being reintroduced if possible [78]. Given the similarities in therapeutic decision-making for leprosy, management in patients with AHPs should be based on the safety profile discussed earlier. Table 2 presents a summary of current evidence on the safety profiles of drugs used in the treatment of mycobacterial infections (tuberculosis and leprosy) in patients with AHPs.

## 5. Treatment of Systemic Fungal Infections

Systemic fungal infections represent serious and potentially fatal complications in patients with underlying conditions related to acquired or primary immunosuppression. Due to the severity of clinical presentations, which primarily involve pulmonary, neurological, cutaneous, and otorhinolaryngological manifestations, it is imperative to use antifungal therapies with the best potential for complete resolution of the infectious process [79,80]. The porphyrinogenic potential of known antifungal agents, such as griseofulvin, has been described in the medical literature for nearly 60 years [81,82]. Whenever topical treatments are considered clinically appropriate, they should be prioritized in patients with AHPs due to the better overall safety profile of topical drugs compared to those metabolized systemically.

In patients with AHPs, fungal infections may present similarly to those seen in other clinical contexts. According to the researchers at The Porphyria Information Centre at the University of Cape Town [78] (available at: URL (accessed on 10 December 2024): https://porphyria.uct.ac.za/porphyria-professionals/porphyria-professionals/prescribing-porphyria-treatment-specific-disorders-poprhyria/management-hiv-patients-porphyria), for the treatment of *Pneumocystis jirovecii* pneumonia, the use of atovaquone or pentamidine is preferred over other antimicrobial and antifungal agents with higher porphyrinogenic risks, such as cotrimoxazole (sulfamethoxazole/trimethoprim), clindamycin/primaquine, and dapsone. Neurocryptococcosis cases in patients with AHPs may be properly treated with intravenous amphotericin B, as well as in cases of esophageal candidiasis or severe oral candidiasis, where intravenous amphotericin B can also be used [78]. Mild local infection presentations, such as uncomplicated oral candidiasis, can also be treated with topical antifungal agents that have a better porphyrinogenic risk profile. Systemic azoles should be avoided in patients with AHPs whenever possible due to their high porphyrinogenic risk. Other aspects of treatment for patients with AHPs, who also have HIV/AIDS, are discussed in more detail in Section 7 of this manuscript. Table 3 presents a summary of the key data regarding the safety profile of antifungals potentially used in the treatment of fungal infections in patients with AHPs.

## 6. Treatment of Parasitic Infections/Helminthiases

The choice of the main antiparasitic drugs fundamentally depends on the type of infectious context and the parasite that has been identified or is suspected. In the case of *Entamoeba histolytica* infection, in patients without AHPs, agents such as metronidazole or tinidazole may be used. For infections caused by *Giardia lamblia*, metronidazole, tinidazole, or albendazole are possible treatments. In acute *Toxoplasma gondii* infections, a combination of sulfadiazine and pyrimethamine may be used. For *Plasmodium* spp. infections in uncomplicated malaria, agents like artemisinin (artemether, artesunate), sometimes combined with lumefantrine, and chloroquine can be used. In resistant malaria forms, a combination of atovaquone with proguanil or quinine with doxycycline or clindamycin may be recommended in patients without AHPs. For schistosomiasis, praziquantel is commonly used. In cases of taeniasis, praziquantel is also the treatment of choice, while albendazole is used for cysticercosis. Infections caused by *Trichuris trichiura*, *Ascaris lumbricoides*, and *Enterobius vermicularis* can be treated with albendazole or mebendazole. For filariasis caused by *Wuchereria bancrofti*, diethylcarbamazine is typically used [83].

In patients with AHPs who require treatment for uncomplicated malaria, quinine or a combination therapy of atovaquone with proguanil can be used, similar to the treatment regimen for resistant forms in individuals without AHPs. According to recommendations from the group of researchers from The Porphyria Information Centre at the University of Cape Town [78] (available at: URL (accessed on 10 December 2024): https://porphyria.uct.ac.za/porphyria-professionals/porphyria-professionals/prescribing-porphyria-treatment-specific-disorders-poprhyria/malaria-prophylaxis-patients-porphyria-travelling-southern-africa), mefloquine, chloroquine, or primaquine can also be used in patients with AHPs; however, the combination of artemisinin with lumefantrine has not yet been fully established in terms of safety for patients with AHPs, and its use should be reserved for extreme situations [84]. On the other hand, the use of dapsone and sulfadoxine is not recommended in clinical practice for patients with AHPs. Routine use of doxycycline, clindamycin, and tetracycline in drug combinations is also not indicated due to safety concerns in patients with AHPs [78]. Decisions regarding the best drug options for the treatment of different parasitic infections should be made on an individual basis, using the safety profile of the various drugs, the clinical stability of the AHP context, and the sensitivity profile of the parasites as the main parameters. Table 4 presents a summary of current evidence regarding the safety profiles of the main agents used in the treatment of human parasitic infections in patients with AHPs.

## 7. Treatment of HIV/AIDS

The treatment of HIV/AIDS using the various available therapeutic options has always represented a significant challenge in clinical practice, both due to the potential for HIV itself to act as a mechanism of clinical decompensation in patients with AHPs and the complexity involved in the metabolism of different active compounds, which are often correlated with porphyrinogenic potential [85]. Similarly, the need for chronic medication treatment for both clinical conditions can, itself, facilitate the occurrence of potential drug interactions [86]. In this context, it is also possible that HIV infection during seroconversion may act as a decompensating mechanism in patients with previously undiagnosed AHPs [85]. Previous case reports have correlated the onset of symptoms or clinical signs of AHPs and other forms of porphyria after exposure to different components of highly active antiretroviral therapy (HAART), such as indinavir [87], efavirenz [88,89], nevirapine [90], and tenofovir [86]. It should also be noted that the need for prophylactic therapies to specific polytherapy for various opportunistic infections (e.g., tuberculosis, systemic fungal infections, *Pneumocystis jirovecii* pneumonia, shingles by Varicella-zoster virus) is a factor requiring greater caution in the treatment of patients with AHPs and HIV/AIDS infection.

Chronic and repetitive exposure to various porphyrinogenic agents in this context, as well as acute exposure and those resulting from therapeutic combinations or substitutions, is believed to pose a higher risk of harm to patients with AHPs and HIV/AIDS. However, the specific set of factors contributing to the increased rate of complications from drug interactions involving porphyrinogenic drugs in some individuals—while others remain relatively stable without clinical or laboratory deterioration—remains unknown. It is recommended in all cases to minimize individual exposure to other porphyrinogenic triggers.

Currently, there is a trend toward initiating HAART in HIV-infected patients at increasingly earlier stages, characterized by higher CD4 count values, less compromised immune function, and fewer associated clinical complications. As a general rule, in patients without a need for treatment of associated opportunistic complications, HAART should be introduced as early as possible when formally indicated. However, a temporary delay in initiating HAART should be considered in AHP patients diagnosed with HIV/AIDS who are experiencing severe and acute opportunistic complications, such as tuberculosis and systemic fungal infections. In such cases, the increased porphyrinogenic potential of combined antimicrobial and HAART therapies could present a higher risk of clinical decompensation of AHPs, which is a key factor in this decision-making process in assessing the current potential for clinical decompensation of AHP in each individual, alongside the clinical severity of both the systemic infection and HIV/AIDS itself [91].

It is highly recommended that patients with AHPs and chronic HIV/AIDS infections receive specialized multidisciplinary care, preferably at outpatient clinics associated with universities, research centers, tertiary centers, or groups experienced in the clinical management of AHPs. If HAART initiation is chosen, there is currently no indication for a gradual or sequential introduction of individual components of antiretroviral therapy. According to recommendations from The Porphyria Information Centre at the University of Cape Town [78] (available at: URL (accessed on 10 December 2024): https://porphyria.uct.ac.za/porphyria-professionals/porphyria-professionals/prescribing-porphyria-treatment-specific-disorders-poprhyria/management-hiv-patients-porphyria), within the possible HAART regimens, consideration should be given to the use of nucleoside reverse transcriptase inhibitors (NRTIs), preferably stavudine with lamivudine, in combination with non-nucleoside reverse transcriptase inhibitors (NNRTIs) or protease inhibitors (PIs). For example, the combination of saquinavir with low-dose ritonavir or lopinavir with low-dose ritonavir may be used. As previously discussed, the use of efavirenz [88,89] and nevirapine [90] has been implicated in the occurrence of acute neurovisceral crisis. These drugs can be considered alternative therapeutic options in cases of adverse events with other NNRTIs and PIs. In cases where therapeutic failure criteria are met, combinations such as abacavir and didanosine with saquinavir with low-dose ritonavir or lopinavir with low-dose ritonavir may be used.

Additionally, according to The Porphyria Information Centre at the University of Cape Town [78] (available at: URL (accessed on 10 December 2024): https://porphyria.uct.ac.za/porphyria-professionals/porphyria-professionals/prescribing-porphyria-treatment-specific-disorders-poprhyria/management-hiv-patients-porphyria), when a clinical suspicion followed by laboratory confirmation of an acute attack arises, it is advisable to suspend antiretroviral therapy. This can involve the immediate discontinuation of all drugs, particularly NNRTIs due to their higher porphyrinogenic risk [78]. However, consideration may be given to temporarily continuing the use of NRTIs and PIs for up to two weeks during the treatment of the acute attack. Re-initiation of antiretroviral therapy should only occur after complete clinical recovery, and it should employ one of the previously discussed HAART regimens associated with a lower porphyrinogenic risk [78]. This approach aims to minimize further complications while maintaining effective HIV management.

## 8. Treatment of Chronic Infections by Hepatitis B and C Viruses

Several factors should be considered when indicating treatment for chronic hepatitis B virus infection, such as viral load, liver complications, and the infectious stage. Pharmacological treatment for chronic hepatitis B infection in patients without AHPs may include the use of nucleoside analogs (such as tenofovir and entecavir) and interferons (such as interferon alpha-2b and peginterferon alpha-2a) [92]. In patients with AHPs and chronic hepatitis B infection, the use of tenofovir and interferon alpha-2a are considered probably not porphyrinogenic, while interferon alpha-2b is considered possibly porphyrinogenic. In such cases, if the treatment is necessary, careful clinical and laboratory monitoring should be performed during the treatment.

In the case of treating chronic hepatitis C virus infection in patients without AHPs, direct-acting antiviral drugs include polymerase inhibitors (e.g., sofosbuvir), entry inhibitors (e.g., elbasvir/grazoprevir, ledipasvir/sofosbuvir), and protease inhibitors (e.g., the combination of glecaprevir/pibrentasvir; the combination of sofosbuvir/velpatasvir/voxilaprevir; the combination of sofosbuvir/daclatasvir). In the decision-making process, knowledge of viral genotypes, resistance profiles, and the presence or absence of liver cirrhosis is essential. In cases of co-infection with hepatitis C virus and HIV, the combination of sofosbuvir/velpatasvir may be used [93]. In the context of patients with AHPs and chronic hepatitis C infection, for most antivirals used, the porphyrinogenic risk profile is not well-established, or there is no conclusive scientific data, such as for sofosbuvir, ledipasvir, elbasvir, glecaprevir, pibrentasvir, velpatasvir, and voxilaprevir. Therefore, it is formally recommended that patients with hepatitis C and AHPs be monitored by a specialized multidisciplinary team to assist in management and clinical and laboratory monitoring.

## 9. Treatment of Influenza A H1N1 and H3N2 Infections and COVID-19

In the treatment of infections caused by the H1N1 or H3N2 influenza viruses in patients without AHPs, oral oseltamivir is commonly used, with zanamivir (inhaled) being used less frequently. In more severe clinical forms, intravenous peramivir may be administered. Specific pharmacological therapy for influenza viruses is especially indicated for individuals with conditions associated with relative immunosuppression, chronic diseases, the elderly, and pregnancy [94]. In cases of moderate or severe COVID-19 infections, intravenous remdesivir, oral molnupiravir, and the combination of oral nirmatrelvir/ritonavir can be used. Tocilizumab or sarilumab may be added in severe cases of the disease [95].

In the context of patients with AHPs presenting with H1N1 or H3N2 influenza virus infections, the use of oseltamivir has shown a good safety profile in various scenarios, although rare descriptions correlate it with exacerbations after recent infectious episodes [96]. A summary of the safety profiles observed for the main antivirals and antiretrovirals [97] used to treat clinical complications and comorbidities in patients with AHPs is provided in Table 5.

## 10. General Safety Profile of Key Antimicrobials in Clinical Practice Used by Patients with AHPs

The general safety profile associated with the use of key antimicrobial agents during the treatment of patients with AHPs has been described throughout the sections of this manuscript for various clinical contexts observed in practice. It is important to emphasize that new knowledge regarding the safety profile and applicability of these drugs in treating different clinical scenarios in AHP patients will continue to emerge, necessitating ongoing review and updates to the data presented at the time of this manuscript’s drafting. It is advisable for medical readers to complement the concepts and knowledge presented here with updated information from at least two of the following primary databases (due to possible conflicting evidence), as discussed earlier [98]: the Drug Database from the American Porphyria Foundation [54], the Acute Porphyria Drug Database by The Norwegian Porphyria Centre/NAPOS [55], and the Safe List from the United Kingdom Porphyria Medicines Information Service and Cardiff Porphyria Service) [56].

The safety profile related to the most common therapies used in the treatment of AHPs must also be considered in the context of potential drug interaction with antimicrobial drugs [99,100]. Givosiran represents a small-interfering RNA therapy which can lead to marked ALA synthase 1 (ALAS1) reduction in the liver [100]. One important aspect to be highlighted is the weak effect of givosiran on cytochrome P450 enzyme activity in the liver, presenting with moderate reduction in CYP1A2 and CYP2D6 activities and minor weak effects on CYP3A4, CYP2C19, and CYP2C9 [100]. Givosiran has no significant known interactions with the most common antibiotics (described in Table 1 and Table 2) and antifungal (Table 3) drugs. Hemin therapies also have the potential to reduce ALA synthase activity [25], and there is currently no evidence of significant drug interactions of hemin with the most common antibiotics and antifungal agents previously described (Table 1, Table 2 and Table 3). It is recommended that the reader, in the near future, check for new updated data on the drug interaction profile for the previously mentioned drugs in relation to the use of givosiran and hemin (including data reported in their product leaflet and pharmacovigilance data), due to the knowledge still under construction in this area of Pharmacology. Table 6 aims to provide a summary with a practical approach suggestion for clinicians in making therapeutic decisions for the treatment of infectious complications (with or without an already established etiological agent) in patients with AHPs.

## 11. Conclusions

The treatment of chronic and acute disease in patients with AHPs requires a series of precautions related to the management and prescription, with treatment decisions always needing to be individualized. Acute and chronic infectious conditions pose significant challenges for clinicians in the follow-up of patients with AHPs, due both to the potential for acute exacerbation or induction of acute neurovisceral crisis by the infectious process itself and the possibility of clinical worsening due to drugs associated with the therapeutic regimen used. It is essential that a specialized care network be involved in the treatment of acute and chronic infectious complications in patients with AHPs, including different specialists in AHPs, infectious diseases, and other relevant specialties.

## Figures and Tables

**Table 1:** Safety profiles of the main drugs used in the treatment of acute bacterial infections in patients with AHPs, according to the Acute Porphyria Drug Database/NAPOS [55] (last accessed on 10 December 2024). Legend: CYP: cytochrome P450.

Antimicrobial Therapy	Porphyrinogenicity/Safety
**Penicillins**	
Benzylpenicillin (G)	Not porphyrinogenic.
Aminopenicillins	
-Amoxicillin	Not porphyrinogenic.
-Amoxicillin/Clavulanate	Not porphyrinogenic. No CYP interaction.
-Ampicillin	Not porphyrinogenic.
Antipseudomonal penicillins	
-Piperacillin	Not porphyrinogenic.
-Piperacillin/Tazobactam	Probably not porphyrinogenic.
Penicillinase-resistant	
-Dicloxacillin	Unknown—prefer to avoid.
-Oxacillin	Unknown—prefer to avoid.
Beta-lactamase inhibitors	
-Clavulanate	Not porphyrinogenic. No CYP interaction.
-Sulbactam	Unknown—prefer to avoid.
-Tazobactam	Unknown—prefer to avoid.
**Cephalosporins**	
Cefadroxil	Probably not porphyrinogenic. No CYP interaction.
Cefaclor	Unknown—prefer to avoid.
Cephalexin	Probably not porphyrinogenic. No CYP interaction.
Cefdinir	Unknown—prefer to avoid.
Ceftazidime	Probably not porphyrinogenic. No CYP interaction.
Cefepime	Probably not porphyrinogenic. No CYP interaction.
Cefprozil	Unknown—prefer to avoid.
Cefotaxime	Probably not porphyrinogenic. No CYP interaction.
Cefiderocol	Unknown—prefer to avoid.
Ceftriaxone	Probably not porphyrinogenic. No CYP interaction.
Ceftazidime	Probably not porphyrinogenic. No CYP interaction.
Cefuroxime	Probably not porphyrinogenic. No CYP interaction.
Ceftaroline	Not porphyrinogenic. No CYP interaction.
Ceftolozane	Unknown—prefer to avoid.
Cefotetan	Unknown—prefer to avoid.
Ceftobiprole	Unknown—prefer to avoid.
**Fluoroquinolones**	
Ciprofloxacin	Probably not porphyrinogenic. CYP inhibitor.
Levofloxacin	Probably not porphyrinogenic. No CYP interaction.
Gemifloxacin	Unknown—prefer to avoid.
Moxifloxacin	Probably not porphyrinogenic. CYP2C9 induction.
Delafloxacin	Unknown—prefer to avoid.
**Macrolides**	
Azithromycin	Probably not porphyrinogenic. Interaction CYP: low.
Clarithromycin	Possibly porphyrinogenic. CYP3A4 inhibition.
Erythromycin	Porphyrinogenic. CYP3A4 inhibition.
Fidaxomicin	Probably not porphyrinogenic. Interaction CYP: low.
**Glycopeptide antibiotic**	
Vancomycin	Not porphyrinogenic.
Dalbavancin	Unknown—prefer to avoid.
Telavancin	Unknown—prefer to avoid.
Oritavancin	Unknown—prefer to avoid.
**Sulfonamides**	
Sulfamethoxazole-trimethoprim	Porphyrinogenic. Sulfonamide induces CYP3A4. Trimethoprim inhibits several CYP.
Sulfasalazine	Probably porphyrinogenic (sulfapyridine).
**Tetracyclines**	
Tetracycline	Probably not porphyrinogenic. Interaction CYP: low.
Doxycycline	Probably not porphyrinogenic. No CYP induction.
Demeclocycline	Probably not porphyrinogenic. No CYP induction.
Eravacycline	Unknown—prefer to avoid.
Minocycline	Probably not porphyrinogenic. No CYP induction.
Omadacycline	Unknown—prefer to avoid.
Sarecycline	Unknown—prefer to avoid.
**Lincomycins**	
Clindamycin	Probably porphyrinogenic. Induction of CYP3A4.
Lincomycin	Unknown—prefer to avoid.
**Rifamycin**	
Rifampicin	Porphyrinogenic. Induction of several CYP.
Rifapentine	Unknown—prefer to avoid.
Rifabutin	Possibly porphyrinogenic. Weak inducer: CYP3A4.
Rifaximin	Unknown—prefer to avoid.
**Aminoglycosides**	
Gentamicin	Not porphyrinogenic.
Amikacin	Not porphyrinogenic.
Tobramycin	Not porphyrinogenic.
**Carbapenems**	
Meropenem	Probably not porphyrinogenic. No CYP interaction.
Imipenem/cilastatin	Probably not porphyrinogenic. No CYP interaction.
Ertapenem	Probably not porphyrinogenic. No CYP interaction.
**Oxazolidinones**	
Linezolid	Probably not porphyrinogenic. No CYP interaction.
Tedizolid	Unknown—prefer to avoid.

**Table 2:** Safety profiles of drugs commonly used in the treatment of mycobacterial infections (e.g., tuberculosis) in patients with AHPs, according to the Acute Porphyria Drug Database/NAPOS [55] (last accessed on 10 December 2024). Legend: CYP: cytochrome P450.

Antimicrobial Therapy	Porphyrinogenicity/Safety
Amikacin	Not porphyrinogenic.
Capreomycin	Unknown—prefer to avoid.
Clarithromycin	Possibly porphyrinogenic. CYP3A4 inhibition.
Clofazimine	Unknown—prefer to avoid.
Cycloserine	Probably not porphyrinogenic. No CYP interaction.
Dapsone	Probably porphyrinogenic. CYP2C9 induction.
Ethambutol	Not porphyrinogenic.
Ethionamide	Unknown—prefer to avoid.
Isoniazid	Possibly porphyrinogenic. Several CYP inhibition.
Kanamycin	Unknown—prefer to avoid.
Levofloxacin	Probably not porphyrinogenic. No CYP interaction.
Linezolid	Probably not porphyrinogenic. No CYP interaction.
Minocycline	Probably not porphyrinogenic. No CYP induction.
Ofloxacin	Probably not porphyrinogenic. No CYP interaction.
Pyrazinamide	Probably not porphyrinogenic. No CYP interaction.
Rifampicin	Porphyrinogenic. Induction of several CYP.
Rifapentine	Unknown—prefer to avoid.
Rifabutin	Possibly porphyrinogenic. Weak inducer: CYP3A4.
Streptomycin	Unknown—prefer to avoid.
Terizidone	Unknown—prefer to avoid.

**Table 3:** Safety profiles of the main drugs used in the treatment of fungal infections in patients with AHPs, according to the Acute Porphyria Drug Database/NAPOS [55] (last accessed on 10 December 2024). Legend: CYP: cytochrome P450.

Antifungal Therapy	Porphyrinogenicity/Safety
**Allylamines**	
-Terbinafine	Probably not porphyrinogenic. No CYP induction.
**Azole**	
-Clotrimazole	Possibly porphyrinogenic. Several CYP induction.
-Fluconazole	Probably porphyrinogenic. CYP3A4 inhibition.
-Isavuconazole	Unknown—prefer to avoid.
-Itraconazole	Probably porphyrinogenic. CYP3A4 inhibition.
-Ketoconazole	Possibly porphyrinogenic.
-Posaconazole	Possibly porphyrinogenic. CYP3A4 inhibition.
-Voriconazole	Probably porphyrinogenic.
**Ciclopirox**	Unknown—prefer to avoid.
**Echinocandins**	
-Anidulafungin	Probably not porphyrinogenic. No CYP induction.
-Caspofungin	Probably not porphyrinogenic.
-Micafungin	Probably not porphyrinogenic. No CYP induction.
**Flucytosine**	Probably not porphyrinogenic.
**Griseofulvin**	Unknown—prefer to avoid.
**Polyene**	
-Amphotericin B	Probably not porphyrinogenic.
-Nystatin	Not porphyrinogenic.

**Table 4:** Safety profiles of the main drugs used in the treatment of parasitic infections in patients with AHPs, according to the Acute Porphyria Drug Database/NAPOS [55] (last accessed on 10 December 2024). Legend: CYP: cytochrome P450.

Antiparasitic Therapy	Porphyrinogenicity/Safety
**Antiamoebics**	
-Chloroquine	Possibly porphyrinogenic. Induction of some CYP.
-Emetine	Unknown—prefer to avoid.
-Metronidazole	Probably not porphyrinogenic.
-Tinidazole	Probably not porphyrinogenic. No CYP induction.
**Anticestodes**	
-Albendazole	Unknown—prefer to avoid.
-Niclosamide	Probably not porphyrinogenic. CYP2D6 inhibition.
-Praziquantel	Unknown—prefer to avoid.
**Antileishmanial**	
-Amphotericin B	Probably not porphyrinogenic.
-Meglumine antimonate	Unknown—prefer to avoid.
-Paromomycin	Probably not porphyrinogenic.
-Pentamidine	Probably not porphyrinogenic.
**Antiprotozoals**	
-Albendazole	Unknown—prefer to avoid.
-Amodiaquine	Unknown—prefer to avoid.
-Artemether/lumefantrine	Probably porphyrinogenic. Inducer: CYP3A4, 2C19
-Atovaquone/proguanil	Probably not porphyrinogenic.
-Chloroquine	Possibly porphyrinogenic. Induction of some CYP.
-Doxycycline	Probably not porphyrinogenic. No CYP induction.
-Furazolidone	Unknown—prefer to avoid.
-Mefloquine	Possibly porphyrinogenic.
-Melarsoprol	Unknown—prefer to avoid.
-Metronidazole	Probably not porphyrinogenic.
-Primaquine	Unknown—prefer to avoid.
-Quinine	Possibly porphyrinogenic. Induction of some CYP.
-Tinidazole	Probably not porphyrinogenic. No CYP induction.
**Antinematodes**	
-Diethylcarbamazine	Unknown—prefer to avoid.
-Ivermectin	Probably not porphyrinogenic.
-Mebendazole	Probably not porphyrinogenic.
-Pyrantel	Unknown—prefer to avoid.
-Tiabendazole	Unknown—prefer to avoid.
**Anti-toxoplasma**	
-Sulfadiazine	Porphyrinogenic. CYP3A4 inducer.
-Pyrimethamine	Unknown—prefer to avoid.
**Anti-trematodes**	
-Praziquantel	Unknown—prefer to avoid.
**Trypanocidal drugs**	
-Benznidazole	Unknown—prefer to avoid.
-Nifurtimox	Unknown—prefer to avoid.
-Pentamidine	Probably not porphyrinogenic.
-Suramin	Unknown—prefer to avoid.
**Broad antiparasitic therapy**	
-Nitazoxanide	Unknown—prefer to avoid.

**Table 5:** Safety profiles of the main drugs used in the treatment of viral and retroviral infections in patients with AHPs, according to the Acute Porphyria Drug Database/NAPOS [55] (last accessed on 10 December 2024). Legend: CYP: cytochrome P450.

Antiviral and Antiretroviral Drugs	Porphyrinogenicity/Safety
Abacavir	Probably not porphyrinogenic. No CYP induction.
Aciclovir	Not porphyrinogenic.
Adefovir Dipivoxil	Probably not porphyrinogenic.
Amantadine	Unknown—prefer to avoid.
Amprenavir	Probably porphyrinogenic. CYP3A4 inducer.
Atazanavir	Probably porphyrinogenic. CYP3A4 inhibitor.
Baloxavir marboxil	Unknown—prefer to avoid.
Boceprevir	Probably porphyrinogenic. CYP3A4 inhibitor.
Brincidofovir	Unknown—prefer to avoid.
Brivudine	Unknown—prefer to avoid.
Cidofovir	Probably not porphyrinogenic. No CYP interaction.
Daclatasvir	Unknown—prefer to avoid.
Dapivirine	Unknown—prefer to avoid.
Darunavir	Possibly porphyrinogenic. CYP3A4 inhibition.
Didanosine	Probably not porphyrinogenic.
Efavirenz	Probably porphyrinogenic. Several CYP induction.
Emtricitabine	Probably not porphyrinogenic.
Etravirine	Possibly porphyrinogenic. CYP3A4 induction.
Famciclovir	Probably not porphyrinogenic.
Favipiravir	Unknown—prefer to avoid.
Foscarnet	Probably not porphyrinogenic.
Ganciclovir	Not porphyrinogenic.
Idoxuridine	Unknown—prefer to avoid.
Indinavir	Possibly porphyrinogenic. CYP3A4 inhibition.
Inosine Pranobex	Unknown—prefer to avoid.
Interferon alfa-2a	Probably not porphyrinogenic.
Interferon alfa-2b	Possibly porphyrinogenic.
Lamivudine	Probably not porphyrinogenic. No CYP induction.
Lopinavir	Probably porphyrinogenic. CYP3A4 inhibition.
Maraviroc	Probably not porphyrinogenic. No CYP induction.
Molnupiravir	Unknown—prefer to avoid.
Nelfinavir	Probably porphyrinogenic.
Nevirapine	Probably porphyrinogenic. CYP3A4/2B6 inducer.
Oseltamivir	Not porphyrinogenic. No CYP interaction.
Remdesivir	Unknown—prefer to avoid.
Ribavirin	Probably not porphyrinogenic. No CYP induction.
Rilpivirine	Probably not porphyrinogenic. CYP3A4 inducer.
Rimantadine	Unknown—prefer to avoid.
Ritonavir	Porphyrinogenic. CYP3A4 inducer/inhibitor.
Saquinavir	Possibly porphyrinogenic. CYP3A4 inhibitor.
Simeprevir	Unknown—prefer to avoid.
Sofosbuvir	Unknown—prefer to avoid.
Stavudine	Possibly porphyrinogenic.
Sunitinib	Probably not porphyrinogenic.
Telbivudine	Probably not porphyrinogenic. No CYP induction.
Tenofovir Disoproxil	Probably not porphyrinogenic. No CYP induction.
Tilorone	Unknown—prefer to avoid.
Tipranavir	Porphyrinogenic. CYP3A4 inducer/inhibitor.
Umifenovir	Unknown—prefer to avoid.
Valaciclovir	Not porphyrinogenic.
Valganciclovir	Not porphyrinogenic.
Velpatasvir	Unknown—prefer to avoid.
Voxilaprevir	Unknown—prefer to avoid.
Zidovudine	Probably not porphyrinogenic.

**Table 6:** Practical Clinical Approach to Decision-Making in the treatment of Infectious complications in patients with AHPs.

Practical Approach to Therapeutic Decision-Making in the Treatment of Comorbidities or Infectious Complications in Patients with AHPs
Initial Step: Recognition of the Current Status of AHPs: (i). Known clinical form (established biochemical and/or genetic diagnosis) or unconfirmed clinical suspicion (e.g., initial clinical suspicion in the first episode of metabolic decompensation); (ii). Clinically stable patient (without acute or chronic complications and with a biochemical profile showing no chronic excretion), stable patient (without acute complications but with occasional chronic complications and a biochemical profile showing variable chronic excretion), patient experiencing an acute neurovisceral crisis, patient with a recent history of acute decompensation (within the last 2–3 months); (iii). Patient currently in outpatient care or hospitalized/emergency care; (iv). Patient using prophylactic maintenance therapies for recurrence prevention (e.g., givosiran) or with access to emergency therapeutic options (e.g., hemin-based therapies) or hospital support (e.g., monitoring in an ICU setting).
2. Sequential Step: Data related to the Infectious Condition: (i). Established infectious clinical condition (e.g., laboratory or radiological evidence, or isolated infectious agent) or isolated clinical suspicion; (ii). Identified infectious agent (or strongly presumed) or possible polymicrobial context (or undefined or not immediately identifiable); (iii). Infectious context: severe acute presentation of a potentially lethal condition (requiring inpatient treatment) or the possibility of outpatient treatment (potentially less severe), presentation of identified chronic infection (outpatient treatment in a planned and safe manner); (iv). Resistance profile of infectious agents to the use of antimicrobials: community-acquired infections, infections in an inpatient or healthcare-associated context, antibiogram available to support decision-making; (v). Clinical condition with specific institutional protocols to be used or national and/or international guidelines/recommendations for the treatment of the infectious complication.
3. Decision-Making—Scenario 1: Severe or potentially immediately lethal infectious complications: opt for the most comprehensive treatment of the systemic infectious context with the highest potential for clinical response, regardless of porphyrinogenic potential; recommend joint evaluation with an AHP specialist and an infectious disease specialist; actively monitor clinical signs and symptoms in an inpatient setting (preferably in an ICU), as well as laboratory evidence of potential exacerbation with acute neurovisceral crisis in patients with AHPs; periodically review the resistance profile of infectious agents where available.
4. Decision-Making—Scenario 2: Infectious complications requiring urgent or planned treatment: always first assess the context of stability or instability of AHPs; (i). if AHP is stable, consider the most appropriate therapeutic regimen for the infectious context that, if possible, has the lowest porphyrinogenic potential; actively advise on warning signs and symptoms of acute neurovisceral crisis; (ii). if AHP is unstable, consider treating the clinical condition in an inpatient setting in an ICU or similar unit with the possibility of periodic clinical and laboratory monitoring, ensuring that signs and symptoms of acute neurovisceral crisis decompensation are properly reassessed; always recommend joint clinical follow-up with an AHP specialist and an infectious disease specialist; periodically review the resistance profile of infectious agents where available (e.g., in cases where there are drugs with a similar or superior efficacy profile and lower porphyrinogenic potential, consider substituting the therapy); always seek the possibility of having rescue therapy available for the treatment of acute neurovisceral crisis (e.g., hemin-based therapy); constantly review the set of drugs used throughout the patient’s treatment.

## Data Availability

All review data generated for this manuscript is presented in the text content and is available to readers upon reasonable request.

## Abbreviations

ADR	Adverse Drug Reaction
AHPs	Acute Hepatic Porphyrias
AIDS	Acquired Immunodeficiency Syndrome
AIP	Acute Intermittent Porphyria
ALA	delta-Aminolevulinic Acid
ALAD	delta-Aminolevulinic Acid dehydratase
ALAS1	ALA synthase 1
APF	American Porphyria Foundation
CAP	Community-Acquired Pneumonia
COVID-19	Coronavirus disease 2019
CYP	cytochrome P450
HAART	Highly Active Antiretroviral Therapy
HCP	Hereditary Coproporphyria
ICU	Intensive Care Unit
MDR-TB	Multidrug-resistant tuberculosis
NAPOS	The Norwegian Porphyria Centre
NAPS	National Acute Porphyria Service
NNRTI	Non-Nucleoside Reverse Transcriptase Inhibitor
NRTI	Nucleoside Reverse Transcriptase Inhibitor
PBG	Porphobilinogen
PI	Protease Inhibitor
PID	Pelvic Inflammatory Disease
MRSA	Methicillin-resistant *Staphylococcus aureus*
SADRs	Serious Adverse Drug Reactions
SAEs	Serious Adverse Events
UKPMIS	United Kingdom Porphyria Medicines Information Service
UTI	Urinary Tract Infection
VP	Variegate Porphyria
WHO-UMC	World Health Organization-Uppsala Monitoring Center
XDR-TB	Extensively drug-resistant Tuberculosis
